# Adenosine Receptor Agonists Increase the Inhibition of Platelet Function by P2Y_12_ Antagonists in a cAMP- and Calcium-Dependent Manner

**DOI:** 10.3390/ph13080177

**Published:** 2020-07-31

**Authors:** Nina Wolska, Hassan Kassassir, Boguslawa Luzak, Cezary Watala, Marcin Rozalski

**Affiliations:** Department of Haemostasis and Haemostatic Disorders, Chair of Biomedical Sciences, Faculty of Health Sciences, Medical University of Lodz, Mazowiecka 6/8, 92-235 Lodz, Poland; n.m.wolska@gmail.com (N.W.); hassan.kassassir1@gmail.com (H.K.); boguslawa.luzak@umed.lodz.pl (B.L.); cezary.watala@umed.lodz.pl (C.W.)

**Keywords:** platelet, adenosine receptor, adenosine receptor agonist, P2Y_12_ antagonist, anti-platelet therapy

## Abstract

We have shown previously that platelet activity can be lowered through the simultaneous inhibition of P2Y_12_ receptor and activation of adenosine receptors (AR). This work explores this concept by testing the antiplatelet potential of nine AR agonists in combination with P2Y_12_ receptor antagonists—cangrelor and prasugrel metabolite. A panel of in vitro methods was used to assess platelet viability, P-selectin expression, GPIIb-IIIa activation, fibrinogen binding, calcium ion mobilization, VASP-P level, and cAMP formation, utilizing whole blood or isolated platelets from healthy volunteers. The AR agonists demonstrated anti-platelet effects, but stimulated signaling pathways to varying degrees. AR agonists and P2Y_12_ antagonists reduced expression of both P-selectin and the activated form of GPIIb-IIIa on platelets; however, the combined systems (AR agonist + P2Y_12_ antagonist) demonstrated stronger effects. The antiplatelet effects of AR when combined with P2Y_12_ were more pronounced with regard to exogenous fibrinogen binding and calcium mobilization. The cAMP levels in both resting and ADPactivated platelets were increased by AR agonist treatment, and more so when combined with P2Y_12_ inhibitor. In conclusion, as AR agonists are fast-acting compounds, the methods detecting early activation events are more suitable for assessing their antiplatelet action. The exogenous fibrinogen binding, calcium mobilisation and cAMP level turned out to be sensitive markers for detecting the inhibition caused by AR agonists alone or in combination with P2Y_12_ receptor antagonists.

## 1. Introduction

The leading cause of death in Western countries, according to current World Health Organisation data, is cardiovascular disease that results primarily from arterial thrombosis dependent on blood platelet hyperactivity. Thromboembolic events can be prevented by anti-platelet therapy [1]. However, currently available therapeutic strategies often demonstrate unsatisfactory safety and efficiency, with one of the key issues being drug resistance stemming from high inter-individual variation among patients [2,3]. Novel platelet inhibitors and/or new therapeutic strategies are needed to provide safe and efficient treatments.

Currently, one of the major targets of antiplatelet drugs is the P2Y_12_ receptor. Its inhibition blocks the ADP-dependent platelet activation pathway [4]—the enhancement of platelet aggregation initiated by another ADP receptor (P2Y_1_), rendering the clot formation process ineffective. Additionally, the inhibition of the third platelet receptor from the P2 class, ATP-gated ion channel receptor P2X_1_, which activation does not directly induce the platelet aggregation but causes fast calcium mobilization and platelet shape change, was also suggested as a potential way of reducing thrombotic events [5]. The most commonly used clinically-approved P2Y_12_ inhibitors are thienopyridines (ticlopidine, clopidogrel, and prasugrel—prodrugs whose short-lived active metabolites are irreversible P2Y_12_ inhibitors), the ATP analogue cangrelor (the first intravenous, reversible, non-competitive P2Y_12_ inhibitor) and the cyclopentyltriazolopyrimidine derivative ticagrelor [2,4].

Efficient anti-platelet treatment is often hindered in clinical practice by reduced sensitivity to many anti-platelet agents and by high inter-individual variation in response to treatment, resulting in bleeding and a high risk of failure. That problem is usually managed by a combined therapy—administering two or more drugs affecting various platelet activation pathways. For example, acetylsalicylic acid (inhibitor of thromboxane A_2_ formation) is often combined with clopidogrel. This approach is however still burdened with a problem of drug resistance, especially in patients suffering from type 2 diabetes—a group with increased risk thromboembolic events [6,7,8].

Adenosine is an important purine metabolite; it is a signalling molecule regulating many cell processes that also serves as a component of nucleic acids and ATP [9,10]. Adenosine receptors (AR) are expressed in many cell types and are involved in a plethora of physiological functions. Structurally, they belong to G protein-coupled receptor family. AR subtypes A_2A_ and A_2B_ are expressed in platelets, while A_1_, and A_3_ are not [9,11]. Platelet AR activation decreases platelet activation and aggregation, mediated by an increase in intracellular cAMP (cyclic adenosine monophosphate) levels [12,13]. Adenosine is a natural AR agonist; however, as it is an extremely short-lasting one (less than 10 s in physiological conditions), there is a great need for synthetic, long-lasting agonists [13,14]. Among synthetic AR agonists, most do not differentiate between A_2A_ or A_2B_ subtypes, but some selective agonists have been identified. AR agonists are believed to block platelet aggregation [15,16,17], and hence interest in their antiplatelet property has been growing [13,18,19,20].

One possible treatment option involves the simultaneous inhibition of the P2Y_12_ receptor and agonization of AR receptors expressed by platelets [13,19]. It appears to be a feasible option [19], and one that may solve the problem of drug resistance [21]. Such an approach could avoid the harmful side effects of anti-platelet treatment associated with high-dose P2Y_12_ inhibitors and provide adequate and consistent antithrombotic protection, regardless of individual responses to low-dose P2Y_12_ antagonists [21].

**Figure 1:**
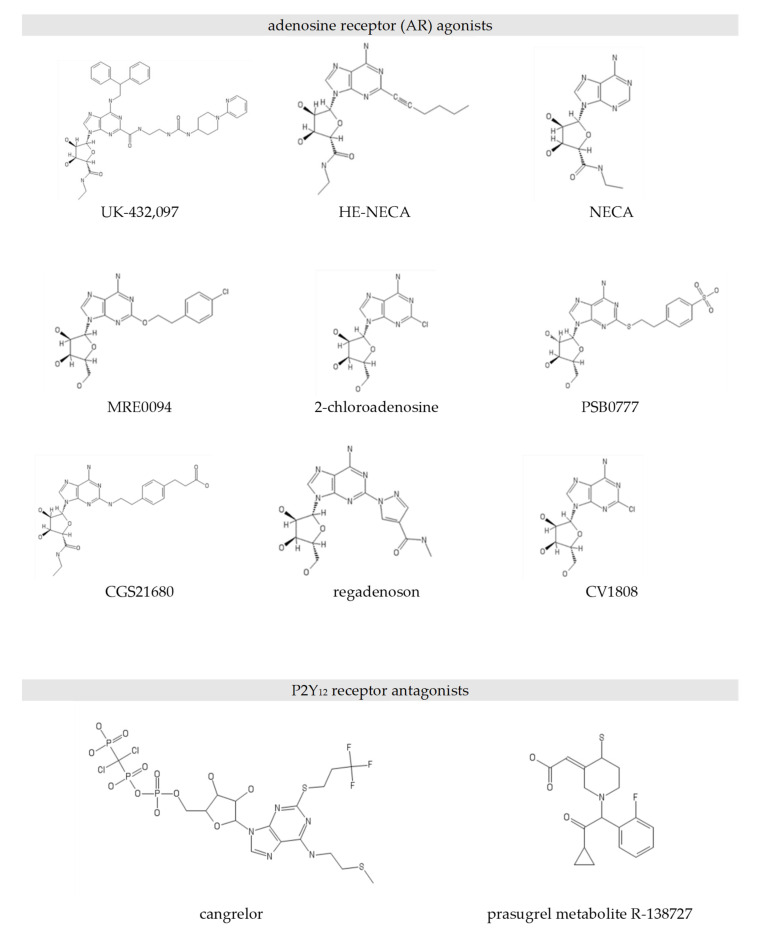
Chemical structures of selected adenosine receptor agonists and P2Y_12_ antagonists.

The aim of the present study is to determine the effectiveness of combined anti-platelet therapy based on a combination of P2Y_12_ inhibitors and adenosine receptor agonists. A set of in vitro methods were used to assess platelet viability, P-selectin expression, GPIIb-IIIa activation, fibrinogen binding, calcium ion mobilisation, VASP [vasodilator-stimulated phosphoprotein] phosphorylation, and cAMP formation. The study examined the anti-platelet effects of two P2Y_12_ inhibitors, cangrelor and prasugrel (active metabolite R-138727), and a panel of nine AR agonists (previously known to inhibit platelet aggregation [11]): PSB0777, CGS21680, MRE0094, 2-chloroadenosine, CV1808, HE-NECA, NECA, regadenoson, and UK423,097 both single (either P2Y_12_ antagonist or AR agonist alone) and dual (simultaneous P2Y_12_ antagonization and AR agonization) models were used (chemical structures of all the compounds tested are shown in Figure 1). It was found that a number of AR agonists are able to inhibit platelet function, as indicated by multiple markers of platelet activation; in addition, AR agonists consistently potentiated the anti-platelet effects of P2Y_12_ inhibitors, including cAMP formation: the pivotal point of platelet signalling.

## 2. Results

AR agonists were used in a combination with two types of P2Y_12_ receptor antagonists: cangrelor (C) or prasugrel metabolite R-138727 (PM), the experimental set up was one AR agonist + one P2Y_12_ antagonist. Each compound was used at its aggregation IC_50_ value [19,21]: PSB0777 23 µM, CGS21680 1 µM, MRE0094 26 µM, 2-chloroadenosine 5 µM, CV1808 25 µM, HE-NECA 0.2 µM, NECA 0.5 µM, regadenoson 1.2 µM, and UK423,097 1 µM, cangrelor 17 nM, and PM 1.3 µM, unless otherwise specified.

Before measuring platelet activity parameters, the potential cytotoxic effects of AR agonists on blood platelets were determined using calcein assay as described previously [22]. The cytotoxicity assay was performed on resting platelets in whole blood preincubated with AR agonists. None of the AR agonists exhibited any cytotoxic effects, indicated by significant changes in cell viability, i.e., the fractions of calcein-negative platelets did not increase compared to untreated controls (Table S1). Significant increase (up to 79%) in the fraction of calcein-negative platelets was observed for the positive control (1% paraformaldehyde) (*p* < 0.005).

Representative cytometric dot-plots and histograms for results described in the Section 2.1, Section 2.2 and Section 2.3 are presented in Figure S1.

### 2.1. The Combined Effect of Adenosine Receptor Agonists and P2Y_12_ Antagonists Increases the Inhibition of P-Selectin Expression

**Figure 2:**
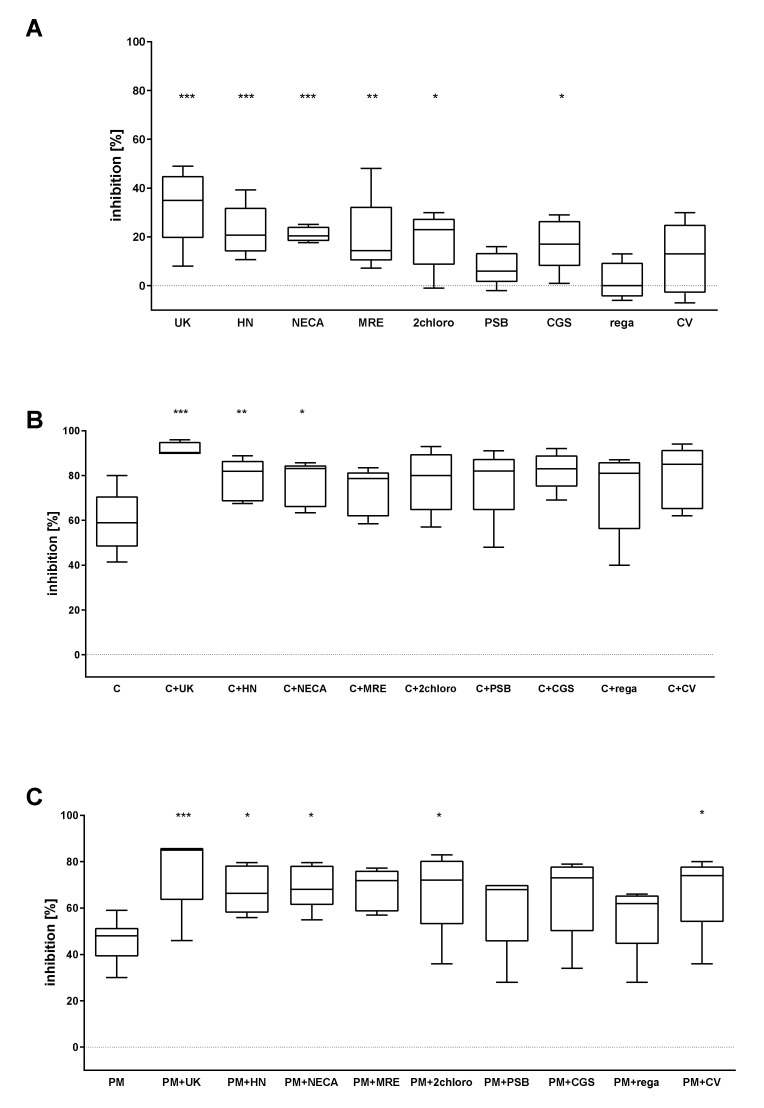
AR agonists intensify the inhibitory effect of P2Y_12_ antagonists on platelet reactivity, as measured by P-selectin expression (*n* = 5). (**A**) Effect of AR agonist on platelets, (**B**) effect on platelets antagonised with the P2Y_12_ inhibitor cangrelor, (**C**) effect on platelets antagonised with the P2Y_12_ inhibitor prasugrel metabolite (PM). Data are presented as median, interquartile range (box), and minimum and maximum values (whiskers). Platelet reactivity was measured after activation with 20 μM ADP, in whole blood. Samples were preincubated at 37 °C for 3 min with AR agonist and cangrelor, or 15 min with PM, all in their previously determined IC_50_. Statistical significance was estimated by one-way ANOVA for repeated measurements with the post hoc Bonferroni’s multiple comparisons test or Friedman’s test with Dunn’s correction for multiple comparisons. Groups containing AR agonists are compared to control samples: untreated samples for AR agonists alone (**A**), P2Y_12_ inhibitor-treated samples for combined systems (**B**,**C**). * *p* < 0.05, ** *p* < 0.01, *** *p* < 0.005. UK—UK423,097, HN—HE-NECA, MRE—MRE0094, 2 chloro -2-chloroadenosine, PSB—PSB0777, CGS—CGS21680, rega—regadenoson, CV—CV1808, C—cangrelor, PM—prasugrel metabolite R-138727 IC_50_ values: UK423,097 1 µM, HE-NECA 0.2 µM, NECA 0.5 µM, MRE0094 26 µM, 2-chloroadenosine 5 µM, PSB0777 23 µM, CGS21680 1 µM, regadenoson 1.2 µM, CV1808 25 µM, and cangrelor 17 nM, and PM 1.3 µM.

The ability of the tested compounds to decrease P-selectin expression, the main surface platelet activation marker, was measured following ADP stimulation. Both P2Y_12_ antagonists used alone significantly reduced platelet activation. The AR agonists UK423,097, HE-NECA, NECA, MRE0094, 2-chloroadenosine, and CGS21680 demonstrated significant inhibition (Figure 2A). More pronounced effects were observed for the following combinations: cangrelor + UK423,097, cangrelor + HE-NECA, cangrelor + NECA (Figure 2B), PM + UK423,097, PM + HE-NECA, PM + NECA, PM + 2-chloroadenosine, and PM + CV1808 (Figure 2C).

### 2.2. The Combined Action of Adenosine Receptor Agonists and P2Y_12_ Antagonists Increases the Inhibition of GPIIb-IIIa Activation and the Inhibition of Fibrinogen Binding

**Figure 3:**
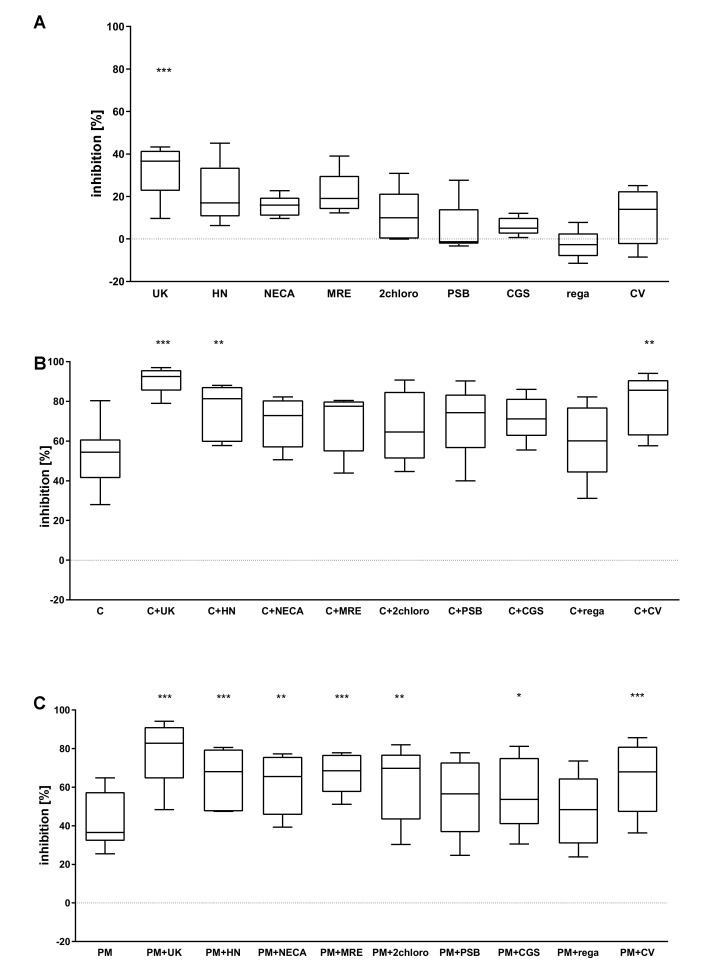
AR agonists intensify the inhibitory effect of P2Y_12_ antagonists on platelet reactivity, as measured by expression of GPIIb-IIIa active form (PAC-1 antibody binding) (*n* = 5). (**A**) Effect of AR agonist on platelets, (**B**) effect on platelets antagonised with the P2Y_12_ inhibitor cangrelor, (**C**) effect on platelets antagonised with the P2Y_12_ inhibitor prasugrel metabolite (PM). Data are shown as median, interquartile range (box), and minimum and maximum values (whiskers). Platelet reactivity was assessed after activation with 20 μM ADP, in whole blood. Samples were preincubated at 37 °C for 3 min with AR agonist and cangrelor, or 15 min with PM, all in their previously determined IC_50_. Statistical significance estimated by one-way ANOVA for repeated measurements with the post hoc Bonferroni’s multiple comparisons test or Friedman’s test with Dunn’s correction for multiple comparisons (groups containing AR agonist are compared to control samples: untreated sample for AR agonists alone (**A**) or P2Y_12_ inhibitor-treated samples for combined version (**B**,**C**)). * *p* < 0.05, ** *p* < 0.01, *** *p* < 0.005. UK—UK423,097, HN—HE-NECA, MRE—MRE0094, 2 chloro—2-chloro- adenosine, PSB—PSB0777, CGS—CGS21680, rega—regadenoson, CV—CV1808, C—cangrelor, PM—prasugrel metabolite R-138727. IC_50_ values: UK423,097 1 µM, HE-NECA 0.2 µM, NECA 0.5 µM, MRE0094 26 µM, 2-chloroadenosine 5 µM, PSB0777 23 µM, CGS21680 1 µM, regadenoson 1.2 µM, CV1808 25 µM, and cangrelor 17 nM, and PM 1.3 µM.

The ability of the tested compounds to decrease GPIIb-IIIa activation in platelets agonized with ADP was measured. Both P2Y_12_ antagonists (Figure 3B,C) and the AR agonist UK 432,097 (Figure 3A) significantly reduced GPIIb-IIIa activation. More pronounced effects were observed for the combined systems cangrelor + UK423,097; cangrelor + HE-NECA (Figure 2B); PM + UK423,097; PM + HE-NECA; PM + NECA; PM + MRE0094; PM + 2-chloroadenosine; PM + CGS21680 and PM + CV (Figure 3B,C).

**Figure 4:**
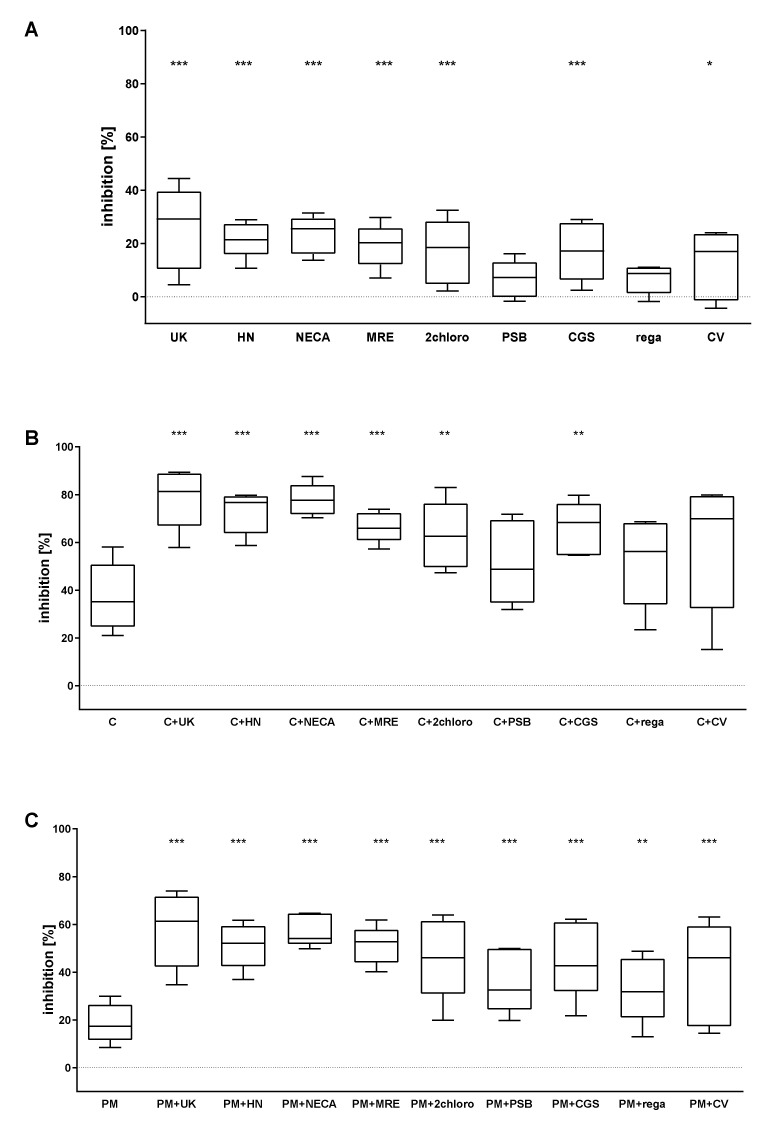
AR agonists intensify the inhibitory effect of P2Y_12_ antagonists on platelet reactivity as measured by exogenous fibrinogen binding (*n* = 5). Panels present data on AR agonist impact on platelets (**A**), platelets antagonised with P2Y_12_ inhibitor: cangrelor (**B**) or prasugrel metabolite (PM) (**C**). Data are shown as median, interquartile range (box), and minimum and maximum values (whiskers). Platelet reactivity was assessed after activation with 20 μM ADP, in whole blood. Samples were preincubated at 37 °C for 3 min with AR agonist and cangrelor or 15 min with PM, all in their previously determined IC_50_. Statistical significance estimated by one-way ANOVA for repeated measurements with the post hoc Bonferroni’s multiple comparisons test or Friedman’s test with Dunn’s correction for multiple comparisons (groups containing AR agonist are compared to control samples: untreated sample for AR agonists alone (**A**) or P2Y_12_ inhibitor-treated samples for combined version (**B**,**C**)). * *p* < 0.05, ** *p* < 0.01, *** *p* < 0.005. UK—UK423,097, HN—HE-NECA, MRE—MRE0094, 2chloro—2-chloroadenosine, PSB—PSB0777, CGS—CGS21680, rega—regadenoson, CV—CV1808, C—cangrelor, PM—prasugrel metabolite R-138727. IC_50_ values: UK423,097 1 µM, HE-NECA 0.2 µM, NECA 0.5 µM, MRE0094 26 µM, 2-chloroadenosine 5 µM, PSB0777 23 µM, CGS21680 1 µM, regadenoson 1.2 µM, CV1808 25 µM, and cangrelor 17 nM, and PM 1.3 µM.

Both P2Y_12_ antagonists significantly reduced platelet activation, as measured by fibrinogen binding (Figure 4B,C). Significant inhibition was also observed for AR agonists: UK423,097, HE-NECA, NECA, MRE0094, 2-chloroadenosine, CGS21680, and CV1808 (Figure 4A).

The following combinations of AR agonists and P2Y_12_ antagonists significantly inhibited the binding of exogenous fibrinogen: cangrelor + UK423,097; cangrelor + HE-NECA; cangrelor + NECA; cangrelor + MRE0094; cangrelor + 2-chloroadenosine; cangrelor + CGS21680 (Figure 4B); PM + UK423,097; PM + HE-NECA; PM + NECA; PM + MRE0094; PM + 2-chloroadenosine; PM + PSB0777; PM + CGS21680; PM + regadenoson; and PM + CV1808 (Figure 4C).

### 2.3. The Combined Action of Adenosine Receptor Agonists and P2Y_12_ Antagonists Increases the Inhibition of Calcium Flux

**Figure 5:**
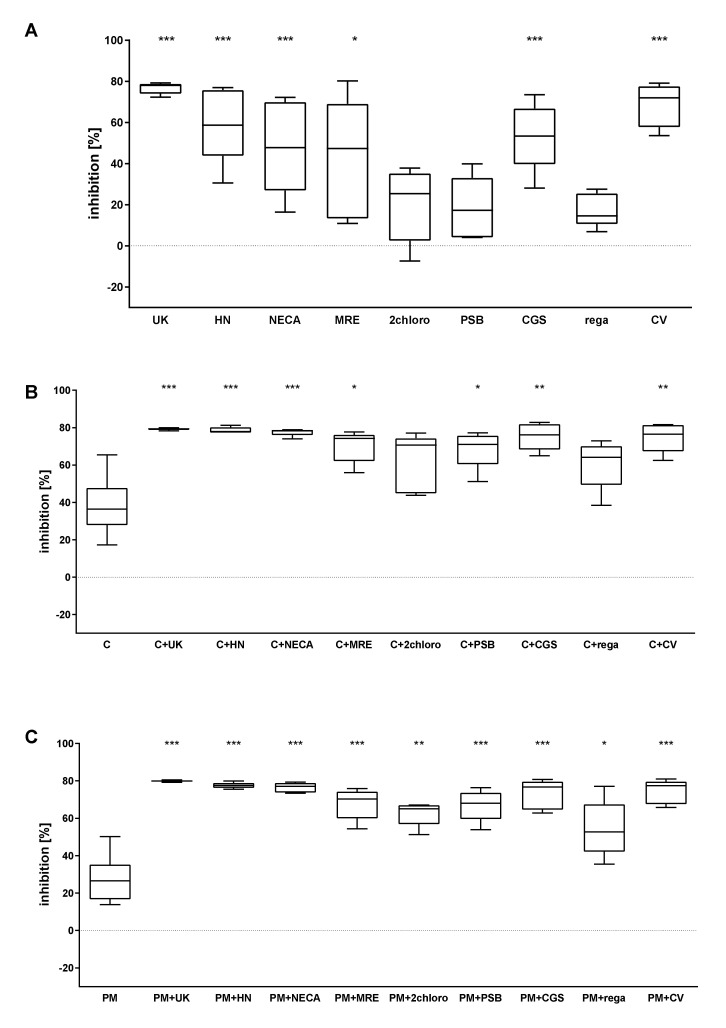
AR agonists strengthen the inhibitory effect on calcium ion mobilisation by of P2Y_12_ antagonists (*n* = 5). Panels present data on the effect of AR agonist on platelets (**A**), platelets antagonised with P2Y_12_ inhibitor: cangrelor (**B**) or prasugrel metabolite (PM) (**C**). Data are presented as median, interquartile range (box), and minimum and maximum values (whiskers). Platelet reactivity was assessed after activation with 20 μM ADP, in whole blood. Samples were preincubated at 37 °C for 3 min with AR agonist and cangrelor, or 15 min with PM, all in their previously determined IC_50_. Statistical significance estimated by one-way ANOVA for repeated measurements with the post hoc Bonferroni’s multiple comparisons test or Friedman’s test with Dunn’s correction for multiple comparisons (groups containing AR agonist are compared to samples: untreated sample for AR agonists alone (**A**) or P2Y_12_ inhibitor-treated samples for combined version (**B**,**C**)). * *p* < 0.05, ** *p* < 0.01, *** *p* < 0.005. UK—UK423,097, HN—HE-NECA, MRE—MRE0094, 2chloro—2-chloroadenosine, PSB—PSB0777, CGS—CGS21680, rega—regadenoson, CV—CV1808, C—cangrelor, PM—prasugrel metabolite R-138727. IC_50_ values: UK423,097 1 µM, HE-NECA 0.2 µM, NECA 0.5 µM, MRE0094 26 µM, 2-chloroadenosine 5 µM, PSB0777 23 µM, CGS21680 1 µM, regadenoson 1.2 µM, CV1808 25 µM, and cangrelor 17 nM, and PM 1.3 µM.

A significant reduction in calcium ion mobilization was shown for cangrelor alone (but not PM) as well as for the samples incubated with AR agonists: UK423,097, HE-NECA, NECA, MRE0094, CGS21680, and CV1808 (Figure 5A). The following pairs demonstrated stronger anti-platelet effects: cangrelor + UK423,097, cangrelor + HE-NECA, cangrelor + NECA, cangrelor + MRE0094, cangrelor + PSB0777, cangrelor + CGS21680, cangrelor + CV1808 (Figure 5B), PM + UK423,097, PM + HE-NECA, PM + NECA, PM + MRE0094, PM + 2-chloroadenosine, PM + PSB0777, PM + CGS21680, PM + regadenoson, and PM + CV1808 (Figure 5C).

### 2.4. The Combined Action of Adenosine Receptor Agonists and P2Y_12_ Antagonists on cAMP Level

The AR agonists and P2Y_12_ inhibitors ability to increase VASP phosphorylation in activated platelets was measured. No significant effects were observed for AR agonist, P2Y_12_ antagonist, or combinations thereof (Table S2). This could be due to the insufficient sensitivity of the applied method. The cAMP measurement was therefore performed to confirm the influence of AR agonists and P2Y_12_ antagonists on this pathway.

Two AR agonists that were established to strongly inhibit platelet functions (UK423,097, and HE-NECA) and one less efficient AR agonist (PSB0777) were used in a combination with P2Y_12_ receptor antagonist cangrelor. Each compound was used in its IC_50_, with the values taken from our previous work (see previous section), or a high dosage (100 µM for AR agonists, 1 µM for cangrelor). Their impact was tested on resting and activated (20 µM ADP) isolated platelets. The results were normalized for platelet count. Positive control of platelets treated with forskolin (5 µM) yielded high results of median 4935→ (interquartile range: 2946, 15586) pmol/1 × 10^8^ plt.

**Figure 6:**
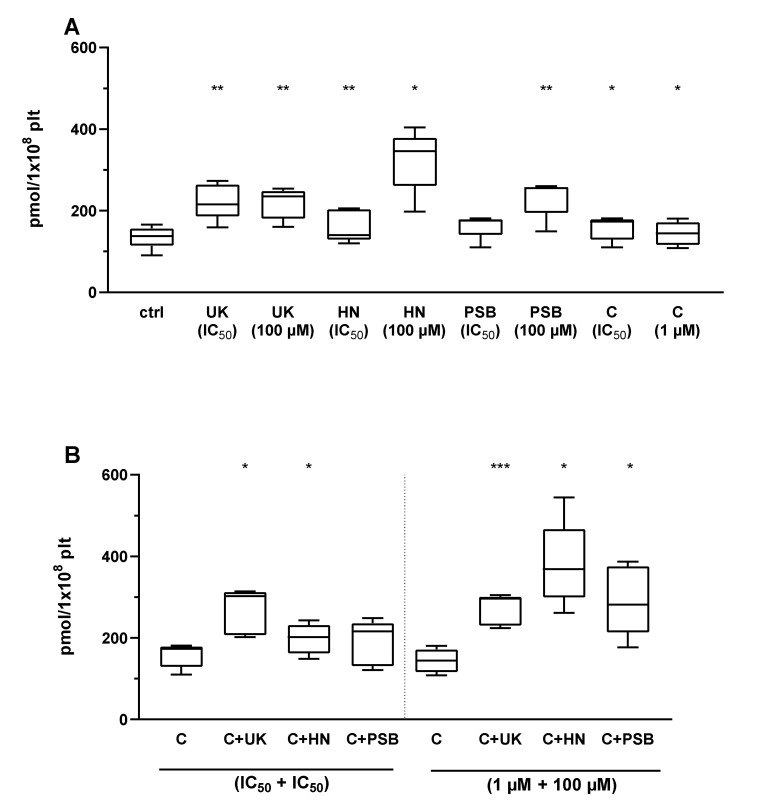
AR agonists strengthen the P2Y_12_ inhibitory effect on platelet reactivity as measured by cAMP formation in resting platelets (*n* = 5). Panels present data on AR agonist impact on platelets in comparison to unstimulated platelets (ctrl) (**A**), platelets antagonised with P2Y_12_ inhibitor: cangrelor (**B**). Data are presented as median, interquartile range (box), and minimum and maximum values (whiskers). cAMP level was measured in isolated platelets. Samples were preincubated at 37 °C for 3 min with AR agonist and cangrelor. Statistical significance estimated by one-way ANOVA for repeated measurements with the post hoc Bonferroni’s multiple comparisons test or Friedman’s test with Dunn’s correction for multiple comparisons (samples containing AR agonist are compared to control samples with P2Y_12_ inhibitor within pertinent group). * *p* < 0.05, ** *p* < 0.01, *** *p* < 0.005. UK—UK423,097, HN—HE-NECA, PSB—PSB0777, C—cangrelor. IC_50_ values: UK423,097 1 µM, HE-NECA 0.2 µM, NECA 0.5 µM, and cangrelor 17 nM.

In resting platelets, the P2Y_12_ antagonist cangrelor significantly increased cAMP formation in both tested concentrations. A significant increase was also found for the AR agonists UK423,097 (both concentrations), HE-NECA (100 µM), and PSB0777 (both concentrations). AR agonists and P2Y_12_ antagonists increased cAMP formation, showing a strong antiplatelet effect, which further increased when use in combination: cangrelor + UK423,097 (both concentration pairs), cangrelor + HE-NECA (both concentration pairs), and cangrelor + PSB0777 (paring in high concentrations) (Figure 6).

**Figure 7:**
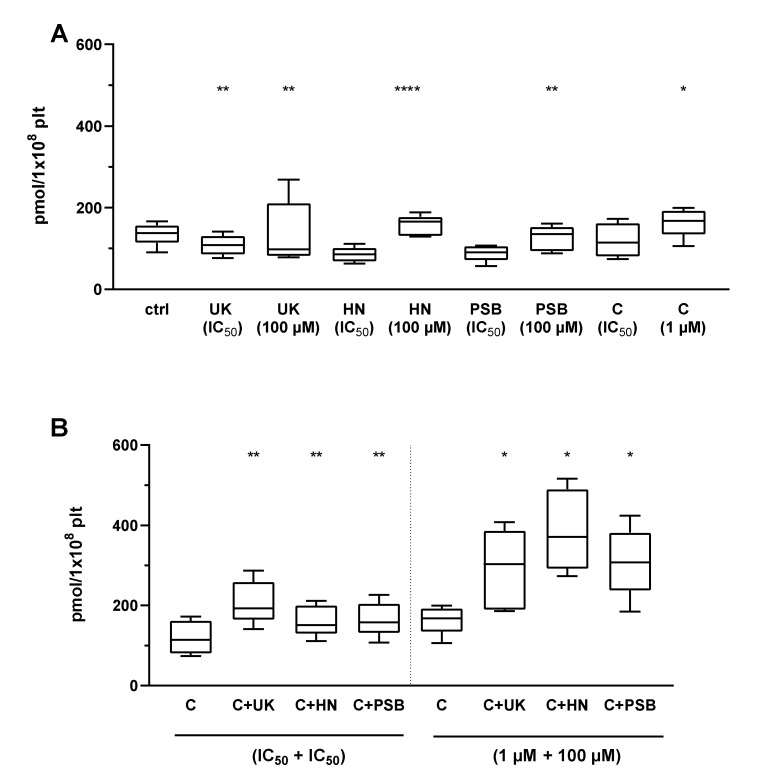
AR agonists strengthen the P2Y_12_ inhibitory effect on platelet reactivity as measured by measured by cAMP formation in ADP- activated platelets (*n* = 5). Figures present data on AR agonist impact on platelets in comparison to platelets stimulated with 20 µM ADP (ctrl) (**A**), platelets antagonised with P2Y_12_ inhibitor: cangrelor (**B**). Data are shown as median, interquartile range (box), and minimum and maximum values (whiskers). cAMP level was measured in isolated platelets after activation with 20 μM ADP. Samples were preincubated at 37 °C for 3 min with AR agonist and cangrelor. Statistical significance estimated by one-way ANOVA for repeated measurements with the post hoc Bonferroni’s multiple comparisons test or Friedman’s test with Dunn’s correction for multiple comparisons (groups containing AR agonist are compared to control group with P2Y_12_ inhibitor) (samples containing AR agonist are compared to control samples with P2Y_12_ inhibitor within pertinent group). * *p* < 0.05, ** *p* < 0.01, **** *p* < 0.001. UK—UK423,097, HN—HE-NECA, PSB—PSB0777, C—cangrelor, IC_50_ values: UK423,097 1 µM, HE-NECA 0.2 µM, NECA 0.5 µM, and cangrelor 17 nM.

In ADP-activated platelets, P2Y_12_ antagonist cangrelor significantly increased cAMP formation in high concentration, as well as AR agonists: UK423,097 (both concentrations), HE-NECA (100 µM), and PSB0777 (100 µM). AR agonists and P2Y_12_ antagonists, when used in combination, deepened the antiplatelets effect compared to these drugs applied alone (Figure 7).

## 3. Discussion

AR agonists are a re-emerging group of compounds with various functions which can be useful in the prevention and treatment of several human diseases [23,24,25]. In the literature, some encouraging results have been published for the application of AR agonists in arrhythmias, cardiac and cerebral ischaemias, neurodegenerative diseases, inflammation, sleep disorders, pain, diabetes, cancer, renal failure as well as glaucoma [26]. Interestingly, agonists of AR receptors expressed on blood platelets (A_2A_ and A_2B_) were previously reported to have remarkable anti-platelet properties [11,13,19,21]. Hypothetically, the agonists of the A_2A_ and A_2B_ adenosine receptors could be a beneficial supplement to current antithrombotic therapy, especially in the light of frequently observed, high inter-individual variability in response to platelet inhibitors.

The antiplatelet effects of adenosine receptor agonists in combination with two P2Y_12_ antagonists (administered intravenously—cangrelor, or orally—prasugrel [27]) were evaluated in this study. We provide further proof of concept and effectiveness of such parings, and investigate molecular pathways most impacted by this combination.

Our previous findings demonstrated the anti-aggregatory activity of some of the AR agonists presented here (UK 432097, MRE 0094, PSB 0777) examined in the absence or presence of the P2Y_12_ antagonists (cangrelor or prasugrel metabolite) [19], and that the use of combination of a P2Y_12_ antagonist and an AR agonist (regadenoson, NECA, and LUF5835) leads to increased inhibition of platelet function than the P2Y_12_ antagonist alone, and that their antiplatelet effect was much more pronounced in individuals with poor response to P2Y_12_ inhibitors [21]. This study supports our recent in vitro work, extending further analyses of the influence of AR agonists, on the effects of the P2Y_12_ antagonists by introducing measurements of a panel of platelet activation hallmarks.

In this work, AR agonists of different selectivity to AR receptors were used—the following agonists were found to be selective for A_2A_ over A_2B_: UK423,097, HE-NECA (also agonizes A_3_ receptor, not expressed on platelets), 2-chloroadenosine (which also agonizes A_1_ receptor, and weakly agonizes A_3_ receptor, both not expressed on platelets), MRE0094, regadenoson, PSB0777, and CGS21680. Other AR agonists investigated in this study (CV1808, and NECA) are not selective between A_2A_ and A_2B_ receptor subtypes. Our previous papers showed that A_2B_ AR receptor selective agonist either did not show anti-platelet effect or had only minimal one, which could be explained by a slight cross-reactivity with A_2A_ AR receptor [13,19]. Even though human A_2A_ and A_2B_ ARs sequence is identical in 59%, no definite proof of a selective A_2B_ AR agonist having antiplatelet properties is available.

Two P2Y_12_ antagonists: cangrelor and prasugrel were utilized to assess whether AR agonists can enhance their antiplatelet effect. Prasugrel is a pro-drug and requires metabolization to its active components, therefore its most abundant stable active metabolite R-138727 was used [28]. P2Y_12_ antagonists, as well as AR agonists, were used at IC_50_ values [19,21]. The relatively high dosage (aggregation IC_50_ = 1.3 µM) required to achieve the effective platelet inhibition most likely stems from the fact that only one of the prasugrel active metabolites is used, whereas in vivo prasugrel metabolization results in a number of active metabolites, all of which may exert an antiplatelet effect of their own. We aimed to reflect suboptimal dosages of both classes of compounds in order to demonstrate the combined effect. In addition, it allowed us to work with concentrations clinically achievable in the patients’ bloodstream.

A_2A_ AR is known as the important receptor on blood platelets and a mediator of the adenosine-dependent inhibition of platelet aggregation [29]. The activation of this receptor leads to the inhibition of internal calcium stores mobilization and external calcium influx, both linked with activation of adenylate cyclase and the increase of cAMP concentration in the cytosol, which leads to the inhibition of platelet activation [30]. The activation of A_2A_ AR was also reported to reduce the P-selectin expression on the platelet cell surface when platelets were stimulated with the use of thromboxane A_2_ or ADP [31]. In this study, we investigated AR agonists at suboptimal concentrations to assess platelet function in vitro at pivotal points of platelet cell signalling—calcium ion mobilization and cAMP formation, also providing proof that they have an ability to further promote the ability of cangrelor and prasugrel metabolite to hinder this process. Similarly, we found that a whole set of markers of platelet activation, such as P-selectin expression, GPIIb-IIIa activation together with fibrinogen binding are reduced by AR agonists alone, and, what is noteworthy, the effects of P2Y_12_ inhibitors on those markers are strengthened by the addition of AR agonists. Our results, therefore, provide further proof that such dual experimental therapy could prevent excessive blood clotting.

**Figure 8:**
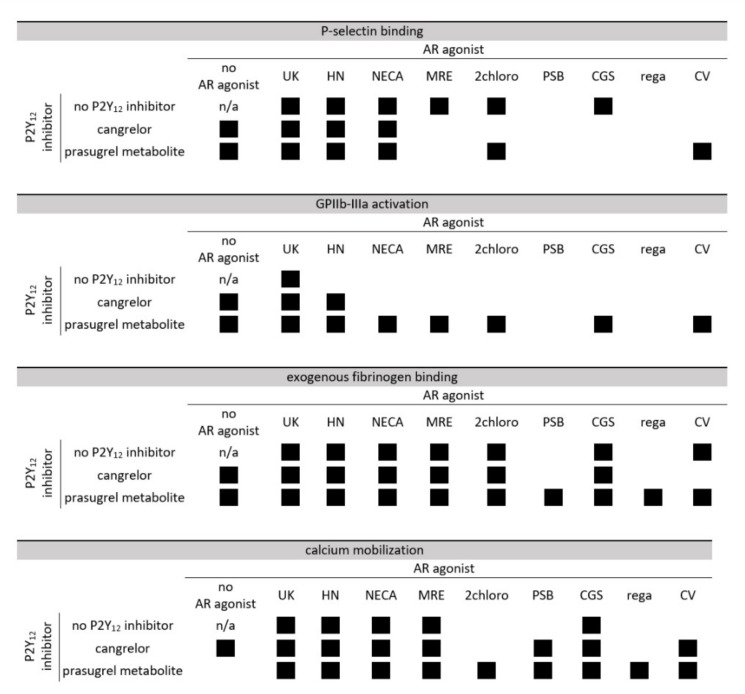
Comparison of AR agonist, P2Y_12_ antagonist or AR agonist and P2Y_12_ antagonist pairing inhibitory effectiveness of ADP-induced platelet reactivity assessed by P-selectin expression, GPIIb-IIIa activation, fibrinogen binding, and calcium ion mobilization. Black boxes indicate statistically significant inhibition for P2Y_12_ antagonist alone and AR agonist alone compared to control non-treated sample, as well for AR agonist and P2Y_12_ antagonist pairs compared to the sample with P2Y_12_ inhibitor only. UK—UK423,097, HN—HE-NECA, MRE—MRE0094, 2chloro—2-chloroadenosine, PSB—PSB0777, CGS—CGS21680, rega—regadenoson, CV—CV1808

This publication provides for the first time the evidence of antiplatelet activity of AR agonist CV1808, demonstrating that CV1808 inhibits exogenous fibrinogen binding, strengthens the antiplatelet effect of PM across all the investigated parameters, and potentiates the ability of cangrelor to limit calcium mobilization. UK423,097 was consistently able to inhibit platelet function across tested parameters, and also HE-NECA and NECA significantly and strongly decreased platelet activation as evidenced by most of the markers (GPIIb-IIIa activation being an exception). On the other hand, regadenoson and PSB0777 did not significantly affect any of the tested platelet activation markers, regadenoson also was unable to strengthen the antiplatelet effect of cangrelor. Overall, however, all the AR agonists studied in this work showed ability to potentiate anti-platelet effect of at least one P2Y_12_ antagonist. A summary of the obtained results is presented in Figure 8.

Interestingly, a high affinity of the A_2A_ receptor agonist does not predict a high anti-platelet effect of this compound. For example, we found a moderate antiplatelet effect for PSB0777 and CGS21680 which have been shown to have the high affinities to A_2A_ receptor (*K*i = 44.4 nM [32], and *K*i = 27 nM [33], respectively), while NECA was observed to have one of the most robust antiplatelet properties, despite its lower affinity to the receptor (*K*i = 620 nM [34]). Similar observations were reported previously [18].

The inter-individual variation in sensitivity to AR agonists was high, especially for weaker agonists, such as PSB0777 or CV1808, the coefficient of variation frequently exceeding 100%. Such a high value and relatively small sample size may account for no significant drop being observed in platelet function in some cases. It is noteworthy that P-selectin expression and GPIIb-IIIa activation methods were found to be less sensitive in detecting the effects of AR agonists in comparison with fibrinogen binding and calcium ion mobilization. P-selectin expression and GPIIb-IIIa activation indicate overall platelet activation and are standard markers of full platelet activation leading to thrombus formation. Additionally, GPIIb-IIIa is a key, final target for anti-platelet therapeutic intervention [35,36]. The changes in those parameters indicate an interference in platelet activation cascade and therefore the inhibition of pro-thrombotic process. Fibrinogen is the main ligand of the GPIIb-IIIa receptor, but activation of this receptor is not synonymous with having bound a molecule of fibrinogen and starting the crosslinking process [37]. In fact, the GPIIb-IIIa activation and fibrinogen binding experimental results are, as expected, highly correlated (*R*^2^ = 0.73), but in our study fibrinogen binding turned out to be a more sensitive marker for detecting the inhibition caused by AR agonists. It could be speculated that binding of exogenous fibrinogen could also be mediated via other platelet-fibrinogen interactions, such as fibrinogen binding to receptors GPVI, GPIb or even non-specific binding. Hence, this parameter could also detect the impact of AR agonists and P2Y_12_ inhibitors on other signalling pathways, but this issue needs further studies.

Calcium mobilization is a very dynamic process, susceptible to rapid change in response to stimulus and therefore a good marker to detect changes in platelet signalling. It governs not only platelet activation, but also secretion and aggregation [38]. It is therefore understandable that subtler changes are possible to register with appropriately sensitive measurement of calcium flux and its inhibition. Generally, as AR agonists are fast-acting compounds, the methods detecting early activation events are more suitable for assessing their antiplatelet action.

cAMP is an important mediator since its increase leads to the activation of signalling pathway (mainly protein kinase A cascade) resulting in the inhibition of platelet function [39]. To assess the effect of AR agonists and their combination with P2Y_12_ antagonists, we have selected two of the most potent AR agonists (UK423,097 and HE-NECA) and one representative of the less effective AR agonists (PSB0777) in combination with cangrelor. The compounds were tested in two concentrations: one being the IC_50_ value and the second one a high concentration selected to demonstrate a maximal inhibitory effect (100 µM for AR agonists and 1 µM for cangrelor); in resting and activated (20 µM ADP) platelets. It was demonstrated that all three tested AR agonists have the ability to increase cAMP formation in human platelets ex vivo (however the weaker one—PSB0777, only in the high concentration of 100 µM). These results are in accordance with the data reported previously in in vitro cell culture studies using standard cell lines such as CHO or HEK-293 [31,40]. The obtained results are also consistent with those previously reported in the literature using human platelets and comparable methods for a different group of AR agonists [18].

Interestingly, this work provides the first report suggesting that the AR agonists alone and in the combination with a P2Y_12_ antagonist increase cAMP formation in ADP-activated platelets. This suggests that such dual experimental therapy may be beneficial in pathologically upregulated platelets or in case of platelets with arrested or delayed cAMP formation rates. The observation that cAMP formation is increased by AR agonists and the fact that AR agonists enhanced the cAMP elevation caused by P2Y_12_ inhibitors confirms that this dual experimental therapy is effective at the pivotal point of platelet activation control.

The phosphorylation of vasodilator-stimulated phosphoprotein (VASP) is a marker of ADP-induced platelet activation through P2Y_12_ receptor [41]. We hypothesised, based on calcium mobilization and cAMP formation results which suggested the inhibition of platelet function through pathway involving VASP phosphorylation, that our proposed dual approach would suppress this process. It seems however, that the applied method (a commercial kit) does not have enough sensitivity to detect changes effected by applied low (subclinical) concentrations of AR agonists and P2Y_12_ inhibitors. Additionally, the method produced inconsistent readings and very high experimental background. In our opinion, therefore, the use of VASP phosphorylation detection kits dedicated to monitoring of P2Y_12_ function in clinical setting is not suitable to research purposes aiming to detect subtle changes in platelet signalling.

The majority of the results (cAMP measurement was an exception) presented in this manuscript were obtained using methods assessing platelet function in whole blood. Such the approach was chosen deliberately since it is known that endogenous adenosine undergoes rapid and excessive uptake and metabolism by erythrocytes [42]. Synthetic AR agonists investigated in this work were reported [13,20,43] to be much more stable in blood, we decided to use the experimental conditions mimicking the physiological ones, and also our aim was to minimize a risk of the incidental platelet activation during the process of the isolation of platelet-rich plasma or platelets.

Our rationale to use methods based on flow cytometry resulted from a need to screen many experimental samples (at least 30 per experiment for each blood donor) providing an opportunity to quickly and reliably obtain a complete panel of data with minimal amount of human material (whole blood) used. Furthermore, flow cytometry analysis is especially valuable when working with blood platelets which are prone to artefactual activation. In the case of cAMP formation experiments, when platelet isolation was inevitable to perform a colorimetric assay, it requires the number of tested combinations to be limited.

## 4. Materials and Methods

### 4.1. Chemicals

Adenosine receptor agonists were purchased from Sigma (St. Louis, MO, USA)—NECA (CAS № 35920-39-9); Cayman (Ann Arbor, MI, USA)—regadenoson (CAS № 313348-27-5), 2-chloroadenosine (CAS № 146-77-0); Tocris Bioscience (Bristol, United Kingdom)—PSB0777 (CAS № 2122196-16-9), CGS21680 (CAS № 124431-80-7), CV1808 (CAS № 53296-10-9); Abcam (Cambridge, UK)—HE-NECA (CAS № 141018-30-6); Axon Medchem (Reston, VA, USA)—UK 432097 (CAS № 380221-63-6)); and MyBioSource (San Diego, CA, USA)—MRE0094 (CAS № 131865-88-8)). Cangrelor (AR-C69931MX) was from Cayman Chemicals). Prasugrel metabolite (R-138727) was obtained from BOC Sciences (Shirley, NY, USA). Calcein AM was obtained from Molecular Probes (Eugene, OR, USA). Antibodies anti-human CD61/PerCP, CD61/PE, CD62P/PE, PAC-1/FITC, mouse IgG1/PE isotype control, mouse IgG1/FITC isotype control, Cellfix, and buffered sodium citrate was purchased from Becton-Dickinson (Franklin Lakes, NJ, USA). Fibrinogen from Human Plasma, Oregon Green 488 Conjugate, and Fluo-4, AM, cell permeant were purchased form Invitrogen (Carlsbad, CA, USA). PLT VASP/P2Y_12_ kit was purchased form BioCytex (Marseille, France). Cell permeant calcium indicator Fluo-4 AM acetoxymethyl ester (Fluo-4 AM), probenecid (water soluble), pluronic acid (water solution) and thapsigargin were purchased from Thermo Fisher Scientific (Waltham, MA, USA). Cyclic AMP Select ELISA Kit was purchased form Cayman Chemical. Phosphate buffered saline pH 7.4 (PBS) was obtained from Corning (New York, NY, USA). Dimethyl sulfoxide (DMSO), adenosine diphosphate (ADP), and bovine serum albumin (BSA) were obtained from Sigma (St. Louis, MO, USA). All other chemicals, unless otherwise stated, were purchased from Avantor Performance Materials Poland S.A. (Gliwice, Poland).

### 4.2. Chemical Solutions Preparation

The stock and working solutions of cangrelor and prasugrel metabolite were prepared in distilled water. The 100 mM stock solutions of AR agonists were prepared in DMSO, excluding PSB0777 which was dissolved in water. Stock solutions were then diluted with DMSO and PBS to working concentrations (as observed by Boncler et al. [19], diluting AR agonist stocks may result in precipitates, which was avoided in this study), and added to the biological material. The dilution factor was chosen to maintain the maximal final concentration of DMSO not exceeding 0.1% in the biological samples in all of the assays. Each compound was used at its aggregation IC_50_ value [19,21]: PSB0777 23 µM, CGS21680 1 µM, MRE0094 26 µM, 2-chloroadenosine 5 µM, CV1808 25 µM, HE-NECA 0.2 µM, NECA 0.5 µM, regadenoson 1.2 µM, and UK423,097 1 µM, cangrelor 17 nM, and PM 1.3 µM, unless otherwise specified.

### 4.3. Blood Donors

Experiments were approved by the Ethics of Research in Human Experimentation Committee at the Medical University of Lodz, approval number (RNN/43/17/KE). Blood was collected from healthy donors who gave written consent (*n* = 34, 30% men and 70% women; mean age 27.5 ± 8.5 years) into a vacuum tube containing 0.105 mol/L buffered sodium citrate (final citrate:blood ratio of 1:9 *v*/*v*) for experiment conducted using whole blood, or into a vacuum tube containing acid citrate dextrose (ACD) (final ACD:blood ratio of 1:7 *v*/*v*) for experiments requiring isolated platelets (cAMP level measurement). All individuals stated that they had not taken medications known to influence platelet function for at least two weeks prior to the study.

### 4.4. Platelet Viability Assay

Platelet viability of resting platelets in the presence of AR agonist was assessed accordingly to Rywaniak et al [22]. Samples were preincubated with AR agonists for 3 min at 37 °C. Positive control (low platelet viability) was blood preincubated in the presence of 1% paraformaldehyde (PFA) for 15 min at 37 °C. Samples were then diluted 10-fold with PBS pH 7.4, labelled with anti-CD61/PE antibodies (15 min, RT) and stained with 0.1 μM calcein-AM (15 min, 37 °C). CD61/PE-positive events (5000) were gathered immediately after staining using flow cytometry (FACSCanto II, BD Bioscience, San Diego, CA, USA). The percentage of calcein-negative platelets was measured.

### 4.5. P-Selectin Expression and GPIIb-IIIa Activation

Whole blood was preincubated with an AR agonist and/or a P2Y_12_ inhibitor for 3 (AR agonists and cangrelor) or 15 (prasugrel metabolite) minutes at 37 °C. Platelets were activated with 20 µM ADP for 5 min at RT. Samples were then diluted 10-fold with PBS, labelled with anti-CD61/PerCP, anti-CD62P/PE and PAC-1/FITC antibodies (15 min, RT), and fixed with CellFix (prepared according to manufacturer instructions) for 1h at RT. Directly before measurement, the samples were diluted 1:1 with PBS and the assay was performed, gathering 10000 CD61/PerCP-positive events, using FACSCanto II flow cytometer (BD Bioscience). The percentage of marker-positive platelets (above isotype cut-off) was measured.

### 4.6. Binding of Exogenous Fibrinogen

Whole blood was preincubated with an AR agonist and/or a P2Y_12_ inhibitor for 3 (AR agonists and cangrelor) or 15 (prasugrel metabolite) minutes at 37 °C. Exogenous Oregon Green-labelled fibrinogen was added to the samples (3 µg/mL), which were subsequently activated with 20 µM ADP for 5 min at RT. Samples were then diluted 10-fold with PBS, labelled with anti-CD61/PE antibodies (15 min, RT), and fixed with CellFix (prepared according to manufacturer instructions) for 1 h at RT. Directly before measurement, the samples were diluted 1:1 with PBS and the assay was performed, gathering 10,000 CD61/PE-positive events, using FACSCanto II flow cytometer (BD Bioscience). The percentage of marker-positive platelets was measured.

### 4.7. VASP-P Measurement

A PLT VASP/P2Y_12_ kit was used to monitor specific platelet ADP receptor antagonists according to manufacturer’s instructions; the analysis was performed on whole blood preincubated with an AR agonist and/or a P2Y_12_ inhibitor for 3 (AR agonists and cangrelor) or 15 (prasugrel metabolite) minutes at 37 °C. Afterwards, samples were supplemented with prostaglandin E1, activated with ADP, fixed, permeabilized and stained with anti VASP-P monoclonal antibodies and next with secondary polyclonal fluorescently labelled antibodies. The non-specific fluorescence was determined using negative isotypic control. Using FACSCanto II flow cytometer (Becton-Dickinson) 10,000 events identified as platelets (CD61-positive) were acquired, and their mean fluorescence intensity was measured using FACSCanto II flow cytometer (BD Bioscience).

### 4.8. Calcium Mobilization

Whole blood was preincubated with an AR agonist and/or a P2Y_12_ inhibitor for 3 (AR agonists and cangrelor) or 15 (prasugrel metabolite) minutes at 37 °C. Next, samples were diluted 10-fold in PBS containing 1 mM of CaCl_2_, and incubated with Fluo-4 AM (final concentration 3 µM) for 15 min at 37 °C, with pluronic acid (final concentration of 0.02%) to facilitate the solubilisation of Fluo-4. To prevent the efflux of calcium indicator out from the cells, samples were supplemented with the inhibitor of organic-anions transporters, probenecid (at final concentration of 2.5 mM). Platelets were then labelled with anti-CD61/PerCp antibodies for 20 min, RT, and samples were diluted 10-fold in PBS containing 1 mM of MgCl_2_. Directly before the measurement platelets were stimulated with ADP (final concentration 20 µM) and after 10 s the end-point fluorescence intensity was measured on FACSCanto II flow cytometer (BD Bioscience).

### 4.9. cAMP

Blood platelets were isolated by a two-step centrifugation at 37 °C: first 200× *g* for 12 min to obtain platelet rich plasma (PRP) with 50 ng/mL PGE_1_ added, and subsequent centrifugation of PRP at 800× *g* for 15 min, again with 50 ng/mL PGE_1_ added, and re-suspension of platelet pellet with Tyrode’s buffer (134 mM NaCl, 12 mM NaHCO_3_, 2.9 mM KCl, 0.34 mM Na_2_HPO_4_, 1 mM MgCl_2_, 10 mM HEPES, 5 mM glucose, pH to 7.4) with 0.3% bovine serum album and 0.5 mM papaverine. Platelet count in the suspension was measured using Sysmex XS-800i (Sysmex, Kobe, Japan) automated morphology instrument. After at least 35 min from last PGE_1_ addition, platelets were incubated with AR agonist and/or P2Y_12_ antagonist for 3 min at 37 °C. If required, platelets were then incubated with 20 µM ADP for 5 min at RT. Subsequently lysis was performed using lysis buffer (50 mM Tris–HCl, 50 mM NaCl, 1 mM MgCl_2_, 1 mMEDTA, 0.1% Triton^®^ X-100, pH 7.4) in 1:1 *v*/*v* ration to the platelet volume for 10 min at RT, and suspension was centrifuged at 10,000× g for 3 min. Cyclic AMP was measured using a Cyclic AMP Select ELISA Kit (Cayman Chemical) according to manufacturer’s instructions.

### 4.10. Statistical Analysis

The results are expressed as median with interquartile range. The Shapiro-Wilk test and Mauchley’s test were used to test the data distribution and sphericity of variances, respectively. Data with Gaussian distribution was analysed with one-way ANOVA for repeated measurements with the *post hoc* Bonferroni’s multiple comparisons test. Data departing from Gaussian distribution were assessed with the Friedman’s test with Dunn’s correction for multiple comparisons. The statistical analysis was performed using the following software packages: Statistica v.13 (Dell Software, Round Rock, TX, USA), and GraphPad Prism (GraphPad Software, San Diego, CA, USA).

## 5. Conclusions

In summary, this work provides the comprehensive evidence of the antiplatelet potential of AR agonists, demonstrated on multiple levels of platelet activation process, from calcium flux inhibition, and cAMP formation increase to restriction of surface markers of platelet activation such as P-selectin expression and GPIIb-IIIa activation together with fibrinogen binding. All the AR agonists studied were able to strengthen the effect of at least one P2Y_12_ receptor inhibitor; therefore, a dual experimental therapy involving combination of P2Y_12_ inhibitors and AR agonists appears to be a feasible solution to overcoming problems of drug resistance leading to dosage increase and resulting in severe side effects, and a way of combating inter-individual variation [21]. The approach combining the blocking of P2Y_12_ receptor and the activation of AR receptors using novel agonists of AR receptors may prove to be a favourable strategy of preventing thrombotic events, and should therefore be further investigated (including in vivo studies in animal models).

## Supplementary Materials

The following are available online at https://www.mdpi.com/1424-8247/13/8/177/s1. Figure S1. Representative cytometric dot-plots and histograms showing dual effect of AR agonist (UK432,097) and P2Y_12_ antagonists (cangrelor and prasugrel metabolite) in whole blood on the expression/binding of platelet surface activation markers and intracellular calcium mobilisation.; Table S1. Effects of AR agonists on platelet viability; Table S2. The effects of adenosine receptor agonists and P2Y_12_ antagonists on VASP phosphorylation.
